# Endoscopic Oncology: Bridging the Interface Between Gastroenterology and Oncology

**DOI:** 10.14309/crj.0000000000000893

**Published:** 2022-11-24

**Authors:** Fredy Nehme

**Affiliations:** 1 Department of Gastroenterology, Hepatology, and Nutrition, The University of Texas MD Anderson Cancer Center, Houston, TX

Cancer is the second most common cause of death in the United States, and gastrointestinal (GI) cancers represent nearly 27% of cancer-related mortality.^1^ In fact, GI malignancies account for more cancers and cancer-related deaths than any other site (Table 1).

**Table 1.** Incidence and mortality of the 5 most common gastrointestinal malignancies (data from surveillance, epidemiology, and end results database 2022)

Site	Incidence (% of all new cancer cases)	Mortality (% of all new cancer deaths)	5-Year relative survival (%)
Colorectum	151,030 (7.9)	52,580 (8.6)	65.1
Pancreas	62,210 (3.2)	49,830 (8.2)	11.5
Liver/bile ducts	41,260 (2.2)	30,520 (5)	20.8
Stomach	26,380 (1.4)	11,090 (1.8)	33.3
Esophagus	20,640 (1.1)	16,410 (2.7)	20.6

Endoscopy has provided physicians with unprecedented access to the GI tract with implications for the management of all major GI malignancies. With rapid advances in endoscopic technology, endoscopists are assuming an increasing role in the procedural treatment of GI malignancies. Applications affect many aspects of cancer management ranging from screening and detection of premalignant and malignant conditions, surveillance of premalignant conditions, staging of cancers, palliation, to definitive therapy of early-stage neoplasms. The wide variety of endoscopic techniques suggest a subspecialty known as endoscopic oncology. As a substantial proportion of endoscopies are currently performed for cancer-related indications, endoscopic oncology pertains to every endoscopist. This article examines the interface between endoscopy and oncology, with focus on the latest endoscopic advances in the diagnosis, management, and treatment of premalignant and malignant GI conditions.

## DETECTION OF PREMALIGNANT AND MALIGNANT LESIONS

Screening endoscopy is a critical tool in preventing GI malignancies. Colonoscopy is the most common endoscopic procedure in the United States and the most sensitive method for colorectal cancer screening.^2^ Extensive data demonstrated the efficacy of colonoscopy in reducing colorectal cancer incidence and mortality.^3^ Over the past several years, there has been increasing recognition of a large variation in the endoscopist's ability to detect and remove precancerous polyps. Therefore, quality assurance programs have been introduced to aid in polyp detection. Multiple innovations have been developed such as enhanced imaging techniques and distal attachments. Artificial intelligence-assisted colonoscopy has been recently made available with promising results.^4^

Esophageal squamous cell carcinoma (ESCC) represents nearly 90% of esophageal cancer cases worldwide, and screening may be beneficial in endemic areas and certain high-risk groups. These include patients with tylosis, lye-induced strictures, and Fanconi anemia.^5^ Endoscopy has been the modality of choice for ESCC screening. Lugol chromoendoscopy is the current standard to highlight areas of abnormality.^6^ Optical enhancement techniques, including narrow band imaging, have also been evaluated in ESCC screening with an improved specificity compared with chromoendoscopy.^7^ Endocytoscopy and high-resolution microendoscopy are also promising for distinguishing between malignant and nonmalignant lesions of the esophagus.

Gastric cancer screening programs have resulted in early gastric cancer (EGC) detection and increased overall survival in areas with high prevalence of disease.^8^ Korean and Japanese guidelines recommend endoscopic screening every 2 years starting at the age of 40 and 50 years, respectively.^8,9^ In addition, endoscopic screening for gastric cancer in high-risk racial and ethnic groups in the United States starting at the age of 50 years and every 3 years was noted to be cost-effective.^10^ The American Society for Gastrointestinal Endoscopy has recommended the consideration of gastric cancer screening among new US immigrants older than 40 years from high-risk endemic regions.^11^

Screening for pancreatic cancer is recommended for certain high-risk individuals with greater than 5% lifetime risk or a 5-fold increased relative risk of pancreatic cancer. These include patients with Peutz-Jeghers syndrome, familial pancreatic cancer, familial atypical multiple mole syndrome, BRACA1/2, PALB2 pathogenic variants, and Lynch syndrome/ataxia telangiectasia mutated pathogenic variant with a first or second-degree relative with pancreatic cancer. When compared with symptom-detected pancreatic cancers, screening-detected cancers have superior outcomes. Guidelines suggest annual endoscopic ultrasound (EUS), magnetic resonance imaging, or a combination of both.^12^

## SURVEILLANCE OF PREMALIGNANT CONDITIONS

Endoscopic follow-up for individuals who are at increased risk of malignancy in whom a preneoplastic lesion has been identified is an important strategy in the management of many GI malignancies. The recognition of Barrett's esophagus as the premalignant precursor to adenocarcinoma has led to societal recommendations for screening endoscopy in individuals with multiple risk factors and surveillance once the diagnosis is established.^13^ Several advances were made to address the limitations of the current screening and surveillance strategies. Established and emerging endoscopic imaging and sampling techniques include chromoendoscopy, confocal laser endomicroscopy, wide-area transepithelial sampling, and volumetric laser endomicroscopy.^13^

Chronic atrophic gastritis and gastric intestinal metaplasia are the 2 main precursors that precede the development of gastric neoplasia. The diagnosis of chronic atrophic gastritis and gastric intestinal metaplasia is currently dependent on histopathology. However, improvements in advanced endoscopic imaging suggest that enhanced imaging can be reliably used to identify premalignant changes and EGC.^14^

Pancreatic cystic lesions are increasingly found in patients undergoing abdominal imaging for unrelated reasons and include non-neoplastic lesions and cystic neoplasms. Pancreatic cystic neoplasms require periodic evaluation with imaging and occasionally EUS to determine cyst morphology in addition to EUS-guided fine needle aspiration of cystic fluid for cytologic, chemical, and molecular studies. Recently, direct optical and endomicroscopic evaluation of pancreatic cysts has become feasible. Real-time in vivo microscopic imaging by needle-based confocal laser endomicroscopy and intracystic biopsy specimens can be obtained with promising results.^15^

## EVALUATION AND STAGING OF NEOPLASMS

Accurate staging is necessary to guide treatment in oncology. In the absence of distant metastasis, EUS plays an important role in the diagnosis and management of GI tumors. It has proven to reduce unnecessary diagnostic and therapeutic procedures, leading to lower morbidity and mortality in cancer treatment.^16^ In addition, fine needle aspiration substantially improves EUS outcomes by enabling tissue sampling, especially suspicious lymph nodes to increase the accuracy of nodal staging. Technologies such as elastography, contrast-enhanced EUS, and high-frequency probes may further improve EUS accuracy. EUS is also a reliable procedure for the evaluation of submucosal lesions of the GI tract, evaluating their layer of origin, echo pattern, and margins.^17^

Endoscopic retrograde cholangiopancreatography (ERCP) is currently the tool of choice for diagnosis and intervention in pancreaticobiliary malignancy. ERCP techniques such as brush cytology and biopsy are performed for the evaluation of biliary strictures. Fluorescence in situ hybridization further improves the yield of biliary brushing cytology samples. Direct visualization of the bile ducts with cholangioscopy is useful in patients with indeterminate biliary lesions and can allow for targeted and accurate tissue sampling. Cholangioscopy may also be useful as a preoperative tool to assess the extent of tumor involvement in cholangiocarcinoma. This can also be used in pancreatoscopy for the visual identification of mucinous neoplasms and assessment of pancreatic strictures with high accuracy for the detection of pancreatic duct neoplasia.^18^

## DEFINITIVE THERAPY OF EARLY-STAGE NEOPLASMS

In the past, the standard approach for the management of gastric cancer was surgical. The first attempt at endoscopic treatment of EGC was reported in 1974.^19^ Endoscopy has since evolved from a purely diagnostic tool to a minimally invasive therapy for malignant conditions. Endoscopic mucosal resection (EMR) was developed in 1984 initially for the treatment of EGC in Japan.^20^ It is currently used for the treatment of esophageal, gastric, small bowel, and colorectal lesions. The main drawback of EMR is the limited size of the specimen that can be resected en bloc. After piecemeal resection, it is difficult to assess the completeness of the resection particularly at the lateral margins. Endoscopic submucosal dissection (ESD) overcomes this limitation, allowing for en bloc resection of certain lesions. ESD has become the standard of care for the management of EGC, as it achieves significantly higher en bloc resection with lower recurrence rates compared with EMR.^21^ Indications for ESD in gastric cancer include nonulcerated EGCs, ulcerated differentiated EGCs < 3 cm, and differentiated EGCs < 3 cm with superficial submucosal invasion.

Most nodular Barrett's esophagus and early Barrett's cancers limited to the mucosa and submucosa (T1sm1) can be treated safely and effectively with endoscopic therapy. Piecemeal EMR using the multiband mucosectomy technique is commonly used for this indication. In addition, there are several effective tools for the eradication of Barrett's mucosa such as radiofrequency ablation (RFA), cryoablation, argon plasma coagulation, and laser ablation. For esophageal squamous cell cancers, ESD is the preferred endoscopic resection technique for many early-stage lesions. Most colonic polyps are small and can be readily managed using conventional polypectomy techniques while 1%–3% may require advanced polypectomy techniques for safe excision.^22^ EMR remains the preferred technique for most of these lesions in the West; however, ESD is having an increasing role as it offers the advantage of a potential cure for low-risk submucosal invasive cancer.

Recently, endoscopic ablation of certain pancreatic tumors has been proposed as an alternative to surgery. EUS-guided RFA for pancreatic neuroendocrine tumors and pancreatic cystic lesions in nonsurgical candidates has shown promising results with a good safety profile.^23^

## PALLIATION OF SYMPTOMS

The goal of endoscopic palliation is to improve patient's symptoms with minimally invasive procedures avoiding surgical intervention. Biliary stenting is commonly used as a palliative measure in unresectable pancreatic cancer or cholangiocarcinoma. Endoscopic RFA is an emerging tool for the palliation of inoperative malignant biliary stenosis used to prolong stent patency.^24^ Malignant luminal obstruction can be relieved throughout the GI tract with esophageal, gastroduodenal, and colonic stenting. A venting gastrostomy may obviate the need for nasogastric suction in those with malignant bowel obstruction not amenable to other therapies. Interventional EUS has emerged as an important option for palliation of GI and pancreaticobiliary malignancies. EUS-guided celiac plexus neurolysis and celiac ganglion RFA provide improved quality of life for patients with pancreatic cancer.^25^ EUS-guided biliary drainage can also be used in malignant biliary obstruction when ERCP is unsuccessful. Approaches include hepaticogastrostomy, antegrade stent placement, choledochoduodenostomy, and rendezvous technique. EUS-guided gastroenterostomy with lumen-apposing metal stent placement is growing in popularity for palliation of malignant gastric outlet obstruction.

Once used mainly to detect large tumors, endoscopy has now become a critical tool in the management of patients with malignancies involving the GI tract. Endoscopic screening and surveillance programs have become an integral part of health care; EUS can provide essential staging information combined with the ability to sample suspicious lesions; and obstruction throughout the GI tract can be palliated. New endoscopic resection and ablative techniques have added to our therapeutic armamentarium in malignant and premalignant conditions with the emergence of therapeutic EUS and ESD. As these technologies continue to develop, the endoscopist has become an integral part of a multidisciplinary effort in the management of patients with GI malignancies.

## DISCLOSURES

Author contributions: F. Nehme: article concept and design, drafting of the manuscript, and is the article guarantor.

Financial disclosure: None to report.
